# No Difference in the Incidence of Complications in Pediatric Patients with Moderate Anemia 30 Days after Pediatric Hip Surgery with and without Blood Transfusion

**DOI:** 10.3390/children9020161

**Published:** 2022-01-27

**Authors:** Phasuth Chutarattanakul, Kamolporn Kaewpornsawan, Jidapa Wongcharoenwatana, Piyanuch Musikachart, Perajit Eamsobhana

**Affiliations:** Department of Orthopaedic Surgery, Faculty of Medicine, Siriraj Hospital, Mahidol University, Bangkok 10700, Thailand; phasuth@gmail.com (P.C.); kamolporn.kae@mahidol.ac.th (K.K.); jidapa.wongcha@gmail.com (J.W.); bangpiya@hotmail.com (P.M.)

**Keywords:** pediatric, hip dysplasia, anemia, complication, osteotomy

## Abstract

This study investigated the association between postoperative blood transfusion and the incidence of postoperative complications 30 days after pediatric hip surgery as well as factors significantly associated with 30-day postoperative complications. Patients were divided into two groups: those with postoperative complications and those with no complications. Postoperative hematocrit (Hct) was categorized as <25%, 25–30%, and >30%. Comparison was made between all postoperative complications at the 30-day follow-up that were influenced by anemia in patients who received transfusion and those who did not. A multivariate logistic regression model was used to identify factors independently associated with postoperative complications. The overall 30-day postoperative complication rate for all patients was 17% (24/138). No significant difference between the transfusion and the non-transfusion patients was found. Preoperative hematocrit (Hct) was significantly lower in the complications group (*p* = 0.030), and both length of stay and 30-day readmission were significantly higher in patients with complications (*p* = 0.011 and *p* < 0.001, respectively). Multivariate analysis revealed female gender (OR: 3.50, 95% CI: 1.18–10.36; *p* = 0.026) and length of hospital stay (OR: 1.23, 95% CI: 1.08–1.41; *p* = 0.004) to be factors independently associated with 30-day postoperative complications. However, no statistically significant difference in the incidence of complications at 30 days following pediatric hip dysplasia surgery was found between patients who received blood transfusion to maintain a Hct level ≥25% and those not receiving transfusion.

## 1. Introduction

Pelvic surgery of pediatric hip dysplasia patients, both with and without proximal femoral osteotomy, is a challenging surgical treatment. The incidence of hip dysplasia in the general population is 1 to 25 per 1000 live births [1,2,3]. Treatment of hip dysplasia in children older than 18 months usually requires open reduction, either with or without osteotomy of the innominate bone and the proximal femur [4].

Pediatric hip surgery, which includes femoral and/or acetabular osteotomy, often results in significant blood loss and postoperative anemia [5]. Postoperative anemia can increase the risk of tissue hypoxia; however, unnecessary transfusion can lead to increased risk of adverse effects, e.g., longer hospital stays and increased medical costs [6]. Additionally, some children need to be transferred to another facility [7]. A range of blood transfusion thresholds has been proposed on the basis of surgical conditions and the presence of underlying disease [8,9], but the decision to transfuse is typically left to the judgement of the surgeon. The purpose of transfusion is to prevent or correct anemia-related problems. Currently, transfusion is recommended in patients with general pediatric illnesses and hematocrit (Hct) levels <21–27% (hemoglobin (Hb) 7–9 g/dL) [10]. More conservative surgeons often decide to transfuse when the Hct level reaches 30% (Hb 10 g/dL).

A recent study of pediatric hip surgery reported that patients who received transfusion required longer hospital stays and experienced more hospital-acquired complications [11]; however, that study did not investigate associations between Hct or Hb level and postoperative complications. Other reports have indicated that moderate postoperative anemia is not associated with increased complications, but those studies were conducted in cardiac, sepsis, and hematologic patients admitted to a pediatric intensive care unit (PICU) [12,13,14,15]. No blood transfusion thresholds have been established for pediatric hip surgery [9]. At our center, we transfuse blood to maintain Hct ≥25% to reduce the risk of postoperative anemia complications. The aims of this study were (1) to investigate the association between postoperative blood transfusion and 30-day postoperative complications after pediatric hip surgery and (2) to identify factors independently associated with 30-day postoperative complications.

## 2. Materials and Methods

Data were collected from January 2009 through to July 2019. Patients were divided into two groups: those with complications after pediatric hip surgery and those without complications. All patients were aged <18 years and had been diagnosed with hip dysplasia on the basis of clinical and radiographic data and required pelvic osteotomy, proximal femoral osteotomy, or combined osteotomy. The exclusion criteria were patients with open reduction of the hip without pelvic or femoral osteotomy, patients with incomplete data, and patients who were lost to follow-up within 30 days postoperatively.

Patient characteristics recorded include gender, age, body mass index (BMI), American Society of Anesthesiologists (ASA) classification, preoperative comorbidities (i.e., cerebral palsy, myelomeningocele, cardiovascular disease, urinary tract problems, and asthma), and hospital readmission within 30 days. Perioperative factors, such as preoperative hematocrit (Hct) levels, reduction in Hct level, estimated level of blood loss (both intraoperative and drainage), and length of stay, were also documented

In our center, the blood transfusion threshold was Hct <25%, while transfusion in patients with Hct ≥25% depended on the symptoms of the patient at the time, e.g., presenting with anemia symptoms such as acute massive hemorrhage, blood pressure ≤90/60 or tachycardia unresponsive to crystalloid or colloid infusion, and congestive heart failure symptoms. For this study, transfusion volume in milliliters per kilogram of body weight (mL/kg) was categorized as either ≥15 mL/kg or <15 mL/kg. Blood transfusion and non-transfusion patients were also categorized on the basis of 24 h postoperative Hct levels into one of three groups: Hct <25%, 25–30%, or >30%.

Thirty-day postoperative complications associated with anemia were recorded, including superficial and deep skin infection, urinary tract infection, pneumonia, systemic bacterial infection, upper respiratory infection, asthma attack, deep vein thrombosis, acute skin reaction, and large hematoma [16,17].

In the comparative analysis, we counted the number of patients who had one or more complications rather than the total number of complications. For instance, if a patient had a superficial infection and an asthma attack, we counted this patient as one in the complication group. In the subgroup analysis of 30-day complications, we reported the number of patients who had one and the number who had two complications. No patients had more than two complications. The incidence of complications was compared between the transfusion and non-transfusion groups among anemic postoperative patients. Complications not associated with postoperative anemia and blood transfusion in the 30-day period after pediatric hip surgery, e.g., nerve injury, recurrent hip dislocation, and malunion or non-union were excluded and not counted in the study.

### Statistical Analysis

The chi-squared test was used to assess statistical significance of the association between groups of non-numerical variables such as gender and comorbidities, and the Mann–Whitney *U*-test was used to assess continuous variables. A multivariable logistic regression model was used to examine the independent effect of risk factors on the incidence of overall 30-day postoperative complications. Each HCT level was investigated using multivariable logistic regression. The results of those analyses are shown as odd ratios (OR) and 95% confidence intervals (CI). A *p*-value < 0.05 was considered statistically significant. SPSS Statistics software was used for all statistical analyses.

## 3. Results

There were 138 patients included in the study, 24 (17%) had complications. Demographic and clinical characteristics of the patients are shown in Table 1. Females were significantly more likely to have complications than males (*p* = 0.044). Most patients were ASA grade I (77.9%) and had no comorbidities (81.2%). The comorbidities identified in the remaining 18.8% of patients included connective tissue diseases (6%), cerebral palsy (3.7%), and myelomeningocele (2.2%). Surgical procedures included pelvic osteotomy or femoral osteotomy (65 patients or 47.1%) and combined femoral and pelvic osteotomies (73 patients or 52.9%). Combined osteotomies had the highest complication rate (16 patients or 21.9%), while 57 pelvic osteotomy or femoral osteotomy patients (87.7%) had no complications. There were 57 cases (78.1%) of complications in combined pelvic and femoral osteotomy patients. 

The preoperative Hct levels were significantly lower in the complications group than in the no complications group (*p* = 0.030) (Table 2). Total blood loss and decline in Hct level were both slightly lower in the complications group, but the difference was not statistically significant. Length of hospital stay and 30-day readmission rates were both significantly higher in patients with complications (*p* = 0.011 and *p* < 0.001, respectively). In the subgroup analysis, postoperative Hct 25–30% and Hct > 30% were not statistically different between the transfused and non-transfused groups (Table 3). Moreover, when we compared blood transfusion by body weight, we found the same lack of statistical significance.

When we evaluated the 30-day postoperative complication incidence for postoperative Hct levels <25%, 25–30%, and >30% in the transfusion and non-transfusion groups, we found no significant difference between the groups for any type of complication, including superficial/deep infection, urinary tract infection, pneumonia, prolonged fever, upper respiratory tract infection, asthmatic attack, deep vein thrombosis, acute allergic reaction from blood transfusion, and large hematoma at the surgical site (Table 4). There were no non-transfused patients in the Hct < 25% group due to the Hct level threshold protocol.

Univariate regression analysis revealed many factors associated with the incidence of complications 30 days after pediatric hip surgery, including gender, age, type of operation, pre- and post-operative Hct level, decreased Hct level, and length of stay. Multivariate analysis that included all of those factors identified only female gender (odds ratio (OR): 3.50, 95% confidence interval (CI): 1.18–10.36; *p* = 0.026) and length of stay (OR: 1.23, 95% CI: 1.08–1.41; *p* = 0.004) as independent predictors of incidence of complications 30 days after pediatric hip surgery (Table 5).

## 4. Discussion

Previous studies have reported an incidence of postoperative complications of 10.3 to 13.1% among hip dysplasia surgery patients [11,18]. The present study found and incidence of 17.1%. The incidence in the present study is slightly higher because we included upper respiratory tract infections (URI) as URI has shown a correlation with anemia [19]. In our study, females had a higher incidence of postoperative complications as they had the majority of cases dysplasia of the hip. A previous study reported females had a greater risk for severe acetabular dysplasia [20] as well as for postoperative complications [11]. Treatment with combined femur and pelvic osteotomies often results in greater blood loss and requires longer operative times than other pediatric hip surgery procedures. However, in this study, we found no association between the type of procedure and the incidence of post-operative complications at 30 days. A future prospective study could provide more information on this issue.

Many previous studies have described comorbidities that can affect the incidence of postoperative complications including cerebral palsy, neuromuscular disease, and pulmonary and cardiac comorbidity [11,16,17,21,22,23]. However, the majority of patients in this study (81%) had no comorbidities, thus limiting the incidence of potentially significant confounding factors that might potentially have affected the association between blood transfusion and the incidence of 30-day postoperative complications. 

Preoperative anemia has been reported to be a statistically significant independent risk factor for postoperative morbidity and mortality in many studies [24,25,26]. In this study, lower preoperative Hct levels were significantly more frequent in the complications group, even though the difference in hematocrit levels between the complications (Hct 36.8%) and non-complications (Hct 38%) groups was only 1.2%. The logistic regression model, however, did not show preoperative Hct levels to be an independent risk factor. At our center, patients receive blood transfusion when their Hct level is <25% or when they present with anemia symptoms such as acute massive hemorrhage, blood pressure ≤90/60 or tachycardia unresponsive to crystalloid or colloid infusion, and congestive heart failure. The local institutional Hct threshold was set at ≥25% to prevent anemia complications; however, a gold standard threshold for Hct and Hb to prevent anemia symptoms has not yet been established. The American Society of Anesthesiologists (ASA) has suggested that transfusion is required when the Hb concentration is <6 g/dL in both adults and pediatric patients [27]. Gunwardana et al. compared two groups of children (Hb 7–10 vs. >10 g/dL) who underwent cleft lip and palate surgery and found no significant differences in perioperative morbidity or recovery rate [28]. A postoperative Hb level of <5 has been suggested as a transfusion threshold in surgery for adolescent idiopathic scoliosis patients [29]. 

Blood transfusion rates in pediatric surgery depends on the type of operation and on the transfusion threshold levels. Sherrod et al. reported an incidence of blood transfusion in pediatric hip surgery of 22%; however, that study included patients who had open reduction without an osteotomy procedure [11]. Yoshihara et al. reported pediatric scoliosis surgery with blood transfusion rates of 18–67% [30,31]. The transfusion rate in our study was 62.3%, as the majority of the operations were combined pelvic and femoral osteotomies.

Large-volume transfusion (>15 mL/kg) may increase the length of hospital stay and the risk of pneumonia [11]. In this study, 15.2% of the patients had a large volume transfusion (>15 mL/kg); however, we did not find transfusion volume to be correlated with the incidence of complications. There were limitations in terms of duration and of frequency in large-volume transfusions in both studies by Sherrod et al. and the present study, and thus prospective analysis is required to control for this factor. We found longer hospital stays to be independently associated with postoperative complications, a result similar to several previous studies [6,7].

Many studies have reported that unnecessary blood transfusion increases the risk of superficial/deep infection, pneumonia, and respiratory complications in orthopedic surgery [32,33] and that it also increases morbidity in pediatric heart surgery [34,35]. However, appropriate blood transfusion is important for treatment of anemia symptoms and the prevention of anemia complications. In this study, all of the Hct <25% patients were transfused following our institution’s protocol. Blood transfusion both in patients with Hct levels of 25–30% and in those with levels of >30% was not significantly correlated with the incidence of 30-day postoperative complications in either the transfused or the non-transfused patients.

A study in a North American registry found an association of perioperative red blood cell transfusions with venous thromboembolism; one case of deep vein thrombosis was reported, and one patient had an acute allergic reaction to a blood component [36].

In the present study, a logistic regression model found that length of stay was statistically significantly associated with the incidence of postoperative complications. In this study, infection was the most frequent complication. Other studies have similarly reported that longer hospital stays are associated with an increased risk of hospital-acquired infection [11,32,33].

### Limitations

This study has some limitations. First, we applied a retrospective study design that limited our ability to make causal inferences. Second, we did not study laboratory investigations related to bleeding risk that could be associated with postoperative anemia, e.g., prothrombin time (PT), partial thromboplastin time (PTT), and international normalized ratio (INR). Third, patient medications were not evaluated, including administration of warfarin and vitamin K, both of which can influence the risk of bleeding. Fourth, operative time was not included as an evaluation parameter due to record inaccuracies. Finally, there was a shortage of postoperative hemoglobin data, and thus we substituted hematocrit level, a variable which played a major role in our analysis.

## 5. Conclusions

Anemia symptoms are the major indication for blood transfusion; however, blood transfusion to maintain Hct at ≥25% does not reduce the incidence of 30-day postoperative complications after a pediatric hip operation. Female gender and length of hospital stay were factors found to be independently associated with the incidence of 30-day postoperative complications. Further prospective study is needed to identify the Hct level at which transfusion can help reduce the incidence of postoperative complications.

## Figures and Tables

**Table 1:** Demographic data.

Characteristic	Total (*n* = 138)	No Complications (*n* = 114)	Complications (*n* = 24)	*p*-Value
Gender (*n*,%)				0.044
Male	66 (47.8%)	59 (89.4%)	7 (10.6%)	
Female	72 (52.2%)	55 (76.4%)	17 (23.6%)	
Mean age (years, (±SD)	7.07 ± 3.8	7.25 ± 3.9	6.21 ± 3.9	0.167
Mean BMI (kg/m^2^, (±SD)	17.8 ± 4.4	17.7 ± 4.2	18.3 ± 5.3	0.711
Pre-operative morbidities (*n*,%)				0.397
No comorbidities	112 (81.2%)	94 (83.9%)	18 (16.1%)	
Comorbidities	26 (18.8%)	20 (76.9%)	6 (23.1%)	
Comorbidities (*n*,%)				
Cerebral palsy	5 (3.7%)	3 (60.0%)	2 (40.0%)	
Myelomenigocele	3 (2.2%)	2 (66.7%)	1 (33.3%)	
Othe neurological disorder	2 (1.5%)	2 (100.0%)	0 (0%)	
Heart disease	0 (0%)	0 (0%)	0 (0%)	
Connective tissue disease	8 (6.0%)	6 (75.0%)	2 (25.0%)	
Urinary system problem	2 (1.5%)	1 (50.0%)	1 (50.0%)	
Hemophilia	1 (0.7%)	1 (100%)	0 (0%)	
Asthma	1 (0.7%)	1 (100%)	0 (0%)	
Two or more comorbidities	2 (1.5%)	2 (100%)	0 (0%)	
ASA grade (*n*,%)				0.324
I	106 (77.9%)	90 (84.9%)	16 (15.1%)	
II	27 (19.7%)	20 (74.1%)	7 (25.9%)	
III	3 (1.5%)	2 (66.7%)	1 (33.3%)	
Type of operation (*n*,%)				0.178
Pelvic osteotomy or femoral osteotomy	65 (47.1%)	57 (87.7%)	8 (12.3%)	
Combined pelvic and femoral osteotomy	73 (52.9%)	57 (78.1%)	16 (21.9%)	

**Table 2:** Hematocrit level, blood transfusion by body weight, mean length of stay, and readmission of patients.

	Total (*n* = 138)	No Complications (*n* = 114)	Complications (*n* = 24)	*p*-Value
Mean pre-operative Hct level (g/dL, ±SD)	37.8 ± 3.1	38.0 ± 3.1	36.8 ± 3.2	0.030
Mean total blood loss (Intraoperative + Drain) (mL, ±SD)	397.4 ± 270.7	401.1 ± 275.5	379.5 ± 251.0	0.768
Mean Hct level drop (g/dL, ±SD)	7.2 ± 5.1	7.4 ± 5.1	5.9 ± 5.2	0.184
Blood transfusion by body weight (mL/kg)				0.341
Non-transfused	52 (37.7%)	46 (85.5%)	6 (11.5%)	
<15	65 (47.1%)	52 (80.0%)	13 (20.0%)	
≥15	21 (15.2%)	16 (76.2%)	5 (23.8%)	
Mean length of stay (days, ±SD)	6.9 ± 5.0	6.1 ± 3.0	10.8 ± 9.0	0.011
30-day readmission	8	2 (25.0%)	6 (75.0%)	<0.001

**Table 3:** Subgroup analysis of incidence of complications between transfusion and non-transfusion groups for different hematocrit levels.

	Complications	No Complications	*p*-Value
Hct < 25%			NA
Transfusion	2 (16.7%)	10 (83.3%)	
No transfusion	0 (0%)	0 (0%)	
Hct 25–30%			0.472
Transfusion	8 (19.5%)	33 (80.5%)	
No transfusion	2 (9.5%)	19 (90.5%)	
Hct > 30%			0.341
Transfusion	8 (24.2%)	25 (75.8%)	
No transfusion	4 (12.9%)	27 (87.1%)	

**Table 4:** Incidence of postoperative complications at 30 days following pediatric hip surgery.

Characteristic	Hct < 25		*p*-Value	Hct 25–30		*p*-Value	Hct > 30		*p*-Value
	Transfusion (*n* = 12)	No Transfusion (*n* = 0)		Transfusion (*n* = 41)	No Transfusion (*n* = 21)		Transfusion (*n* = 33)	No Transfusion (*n* = 31)	
Infection									
- Superficial infection	0	0		1 (2.4%)	0	1.000	4 (12.1%)	1 (3.2%)	0.356
- Deep infection	0	0		3 (7.3%)	0	0.545	0	0	
- Urinary tract infection	0	0		0	2 (9.5%)	0.111	1 (3.0%)	1 (3.2%)	1.000
- Pneumonia	0	0		1 (2.4%)	0	1.000	0	0	
- Prolonged fever	0	0		2 (4.9%)	0	0.545	0	0	
- URI	1 (8.3%)	0		1 (2.4%)	0	1.000	2 (6.1%)	1 (3.2%)	1.000
- Asthma attack	0	0		2 (4.9%)	0	0.545	0	0	
Deep vein thrombosis	0	0		0	0		1 (3.0%)	0	1.000
Acute allergic reaction	1 (8.3%)	0		0	0		0	0	
Surgical site large hematoma	0	0		0	0		0	1 (3.2%)	0.484

Total number of patients with postoperative complications = 24. Patients experiencing one postoperative complication = 21. Patients experiencing two postoperative complications = 3.

**Table 5:** Univariable and multivariable logistic regression analysis of postoperative complications after pediatrics hip surgery.

Predictors	Univariable Analysis		Multivariable Analysis	
	OR (95% CI)	*p*-Value	Adjusted OR (95% CI)	*p*-Value
Gender				
Male	Ref.			
Female	2.61 (1.00, 6.76)	0.049	3.50 (1.18, 10.36)	0.026
Age (years)	0.93 (0.83, 1.05)	0.223		
Type of operation				
Single osteotomy ^1^	Ref.			
Combined osteotomy ^2^	2.00 (0.79, 5.04)	0.142		
Preoperative Hct level (g/dL)	0.88 (0.76, 1.02)	0.091		
Hct ^3^ drop level (g/dL)	0.94 (0.86, 1.03)	0.185		
Transfusion				
No	Ref.			
Yes	2.03 (0.75, 5.50)	0.164	1.29 (0.42, 3.94)	0.657
Postoperative Hct level				
<25%	Ref.			
25–30%	0.96 (0.18, 5.07)	0.963	1.04 (0.18, 6.00)	0.969
>30%	1.15 (0.22, 5.97)	0.864	1.25 (0.22, 7.28)	0.802
Length of stay (days)	1.19 (1.06,1.32)	0.002	1.23 (1.08, 1.41)	0.004

^1^ Femoral osteotomy or pelvic osteotomy; ^2^ combined femoral osteotomy and pelvic osteotomy; ^3^ Hct, hematocrit.

## Data Availability

The datasets used and/or analyzed during the current study are available from the corresponding author on reasonable request. The data are not publicly available because they contain information that could compromise the privacy of the research participants.
